# Origin of Sugar in the Animal Economy

**Published:** 1849-07

**Authors:** 


*Origin of Sugar in the Animal Economy.*—The following conclusions are copied from a memoir on the above subject, by Dr. C. Bernard in the *Archives Générales de Médecine:*

1st. That in the physiological state there exists constantly and normally diabetic sugar in the arterial blood, and in the liver of man and animals.

2d. That this sugar is formed in the liver, and that it is independent of a sugar or starch alimentation.

3d. That the formation of sugar in the liver begins before the animal is born.

4th. That it appears connected with the integrity of the pneumogastric nerves.—D. W. Y.

